# Hexa Helix: Modified Quad Helix Appliance to Correct Anterior and Posterior Crossbites in Mixed Dentition

**DOI:** 10.1155/2012/860385

**Published:** 2012-10-16

**Authors:** Syed Mohammed Yaseen, Ravindranath Acharya

**Affiliations:** ^1^Division of Pediatric Dentistry, Department of Preventive Dental Sciences, College of Dentistry, King Khalid University, P.O. Box 3263, Abha 61471, Saudi Arabia; ^2^Division of Pediatric Dentistry, Pulla Reddy Dental College, G.P.R. Nagar, Nandyal Road, Kurnool 518002, Andhra Pradesh, India

## Abstract

Among the commonly encountered dental irregularities which constitute developing malocclusion is the crossbite. During primary and mixed dentition phase, the crossbite is seen very often and if left untreated during these phases then a simple problem may be transformed into a more complex problem. Different techniques have been used to correct anterior and posterior crossbites in mixed dentition. This case report describes the use of hexa helix, a modified version of quad helix for the management of anterior crossbite and bilateral posterior crossbite in early mixed dentition. Correction was achieved within 15 weeks with no damage to the tooth or the marginal periodontal tissue. The procedure is a simple and effective method for treating anterior and bilateral posterior crossbites simultaneously.

## 1. Introduction

Anterior crossbite is defined as a malocclusion resulting from the lingual positioning of the maxillary anterior teeth in relationship to the mandibular anterior teeth [1]. An anterior crossbite is present when one or more of the upper incisors are in linguo-occlusion (reverse over jet). This may involve just a single tooth or could include all four upper incisors [2]. 

Anterior dental crossbite has a reported incidence of 4-5% and usually becomes evident during the early mixed-dentition phase [3]. A variety of factors has been reported to cause anterior dental crossbite, including a palatal eruption path of the maxillary anterior incisors; trauma to the primary incisor resulting in lingual displacement of the permanent tooth germ; supernumerary anterior teeth; an over-retained necrotic or pulpless deciduous tooth or root; odontomas; crowding in the incisor region; inadequate arch length; and a habit of biting the upper lip. Various treatment methods have been proposed to correct anterior dental crossbite, such as tongue blades, reversed stainless steel crowns, fixed acrylic planes, bonded resin-composite slopes, and removable acrylic appliances with finger springs [4]. 

Posterior crossbite is defined as any abnormal buccal-lingual relation between opposing molars, premolars, or both in centric occlusion [5]. The reported incidence of posterior crossbites ranges from 7% to 23% of the population [6]. The etiology of posterior crossbite includes genetics, environmental factors, and habits. However, it is usually associated with transverse maxillary deficiency. This deficiency is often the result of asymmetric growth of mandible or maxilla, discrepant width of maxilla or mandible, crowding, premature loss, or prolonged retention of primary teeth, impaired nasal breathing, digit sucking, abnormal swallowing habits, and temperomandibular disorders [7]. Treatment options for posterior crossbite correction includes maxillary arch expansion, removal of occlusal interferences, and elimination of functional shift. Maxillary arch expansion can be achieved either by slow maxillary expansion (fixed or removable) or rapid maxillary expansion.

Anterior and posterior crossbites in the early mixed dentition are believed to be transferred from the primary to the permanent dentition and can have long-term effects on the growth and development of the teeth and jaws [8]. Early cross-bite corrections lead to a stable and normal occlusion pattern and contribute to symmetrical condyle growth, harmonious TMJ, and overall growth in the mandible [9]. This case report describes the use of a simple fixed appliance to manage anterior and posterior crossbites in the mixed dentition.

## 2. Case Presentation

**Figure 1:**
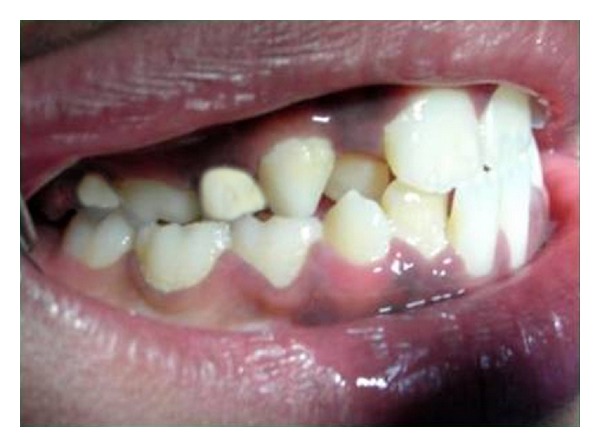
Lateral intraoral view showing anterior and posterior crossbite on the right side.

**Figure 2:**
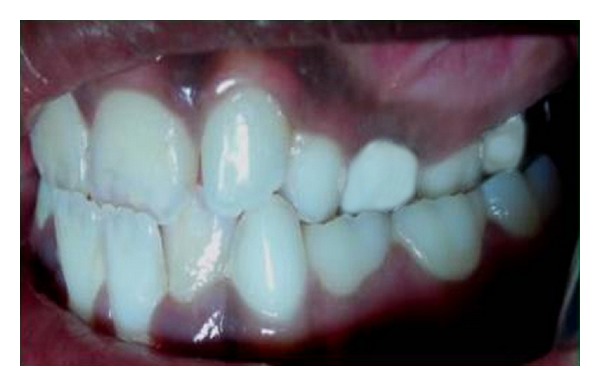
Lateral intraoral view showing anterior and posterior crossbite on the left side.

An 8-year-old girl was referred to the Department of Paediatric dentistry, College of Dental sciences, Davangere for a routine dental check-up. Extraorally, she had a balanced face with a pleasant profile, with the maxillary dental midline coincident with the facial midline. There was no deviation of chin from the facial midline, and the entire maxillary right and left posterior segments were tipped palatally. She presented in the mixed dentition stage with Class I left and half-cusp Class II right molar relationships. An anterior crossbite involving all the maxillary anterior teeth except permanent left lateral incisor, and bilateral posterior crossbite were evident (Figures 1 and 2).

### 2.1. Treatment Plan

An early interceptive treatment approach was essential to alleviate both anterior and posterior crossbite in the above said patient. This can be achieved either with a removable expansion appliance with jack screw or a fixed appliance such as hexa helix. Removable appliances were not preferred in these situations as they tend to get displaced as the turning frequency decreases following activation. Moreover, poor patient compliance with removable appliance can cause relapse of the previous expansion and poor success rate. Therefore, a fixed appliance was chosen.

### 2.2. Appliance Design

The fixed appliance planned was hexa helix; a modification of quad helix in which both anterior and posterior crossbites can get corrected simultaneously. The traditional quad helix consists of a pair of anterior helices and posterior helices. The free wire ends adjacent to the posterior helices are called outer arms. They rest against the lingual surface of the posterior teeth and are soldered on to the lingual aspect of the molar bands. In our case, we incorporated an additional helix to the traditional design on either side of the outer arm. This additional helix was utilized to correct the anterior crossbite.

### 2.3. Clinical Management

**Figure 3:**
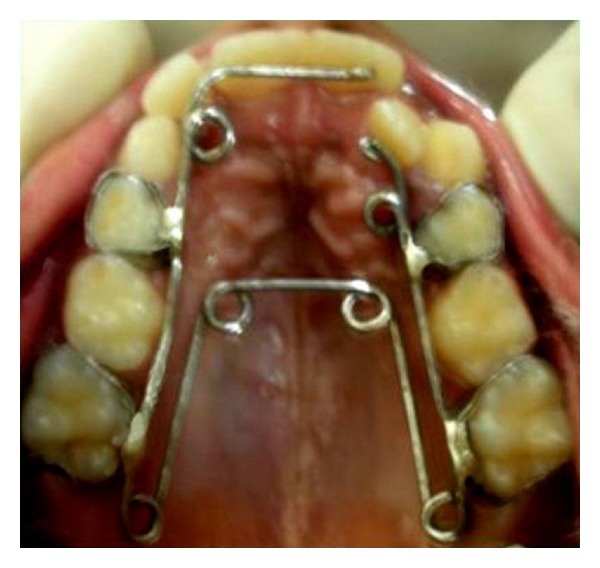
Activated hexa helix appliance.

**Figure 4:**
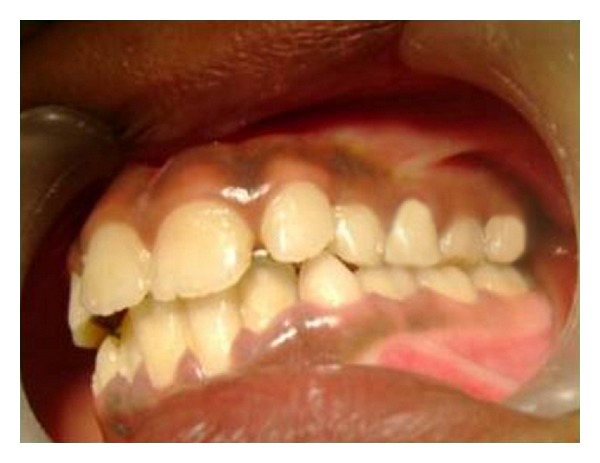
Lateral intraoral view following correction of anterior crossbite and posterior crossbite on the left side.

**Figure 5:**
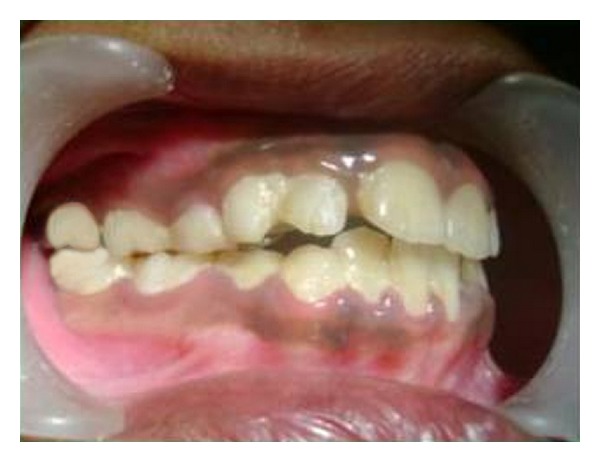
Lateral intraoral view following correction of anterior crossbite and posterior crossbite on the right side.

Orthodontic bands were adapted on either side of maxillary permanent first molars and maxillary primary first molars, followed by fabrication of appliance with 0.036 stainless steel wire as per the above mentioned design. The appliance was activated prior to insertion and then cemented (Figure 3). The helices were activated with a three prong plier once every 3 weeks. A posterior bite plane using glass ionomer cement was placed on the occlusal surfaces of mandibular posterior teeth for the time being to make the bite open anteriorly so that the anterior teeth that are in crossbite can be moved labially, following which they were removed. Within a period of 6 weeks almost all the anterior teeth were corrected out of crossbite except maxillary permanent right lateral incisor as there was not enough sufficient space. Hence selective grinding was done on maxillary right primary canine to make room for lateral incisor. Following this lateral incisor moved labially uneventfully. However most anterior helices were left intact to act as retentive appliance. Regarding posterior teeth, there was a transverse expansion of 5 mm achieved which was sufficient enough to bring the maxillary posterior teeth to normal relation with their mandibular counterpart (Figures 4 and 5) which took approximately 15 weeks. Following this the appliance was left in the mouth for 3 months for retention. Post treatment exhibited class I molar relation. Two years post treatment patient has a functional occlusion without crossbite.

## 3. Discussion

One of the chief objectives of paediatric dentistry is to guide the developing dentition to a state of normalcy in line with the stage of oral-facial growth and development [10]. The period of mixed dentition offers the greatest opportunity for occlusal guidance and interception of malocclusion [11]. If delayed to a later stage of maturity, treatment may become more complicated [12].

The rate of self-correction of crossbites is too low to justify without intervention. Posterior crossbites in the deciduous dentition showed self-correction of between 0% and 9% [6, 13]. Kutin and Hawes [6] reported a spontaneous correction rate of only 8% in their sample of 515 children, 5 to 9 years of age. However, Thilander and coworkers [13] found 21% spontaneous correction of posterior crossbite in a randomized clinical trial of 61 children ages 4 to 13. It is noted that treatment of posterior crossbite in the deciduous dentition period can be realized through the grinding of deciduous teeth that causes premature occlusal contact. The treatment during the mixed dentition period, however, relies on the transversal expansion of the maxillary teeth [14].

White [15] stated that both anterior and posterior crossbites require early correction for functional reasons and the correction of an anterior crossbite is also required for aesthetic reasons. It has been found that dental features were the fourth most common target for teasing, but comments made about teeth were considered to be more hurtful than any other feature especially in the 8–10 year age group [16].

The goal of early treatment is to minimize or eliminate skeletal, dentalveolar, and muscular problems by the end of the transition to the permanent dentition. It has been observed that correction of crossbite in mixed dentition can be successful in 84–100% of cases [17]. Moreover, evidence suggests that a short course of orthodontic treatment in the mixed dentition may improve function and aesthetics, reduce the potential for teasing, and remain relatively stable once the appliance is removed [18].

Fixed appliance treatment was chosen as it provides advantages such as minimal discomfort, reduces need for patient cooperation, better control of tooth movements, and cost effectiveness. Fixed appliances are typically favored for expansion due to reduced cost and treatment time [19]. Ninou and Stephens [20] stated that crossbites with a functional displacement require treatment and that a maxillary fixed appliance is their preferred technique. Moreover removable appliances have various disadvantages such as need for patient cooperation, difficulty in speech/eating decalcification, caries, palatal hyperplasia, fungal infections, and incorrect activation leading to unhelpful results [18]. The increased treatment time and cost for removable expansion plates makes it a poor choice for the current situation [21].

Modified quad helix was preferred in our case to correct both anterior and bilateral posterior crossbite simultaneously for various reasons. Quad-helix appliance can deliver sufficient forces to promote skeletal changes on maxillary bone in younger patients (during deciduous and mixed dentitions phases [22]). Quad-helix is used as an expansion device because it is a very versatile appliance, with applications such as: molar rotation control, torque, and tipping control. It can also produce advancement in the incisor region and create greater anterior expansion, resulting in an improved arch form (taking advantage of the anterior arms that deliver a “sweeping action”). Furthermore the practitioners do not need the patient's or parent's cooperation to reach the set objectives [23]. In general, using the quad helix for treatment leads to skeletal changes in maxillary bone, when desired by the practitioner and indicated in the treatment objectives. Adjustments are made by simply changing the amount and frequency of the activations. It is observed that when correctly employed, the quad helix can produce similar results to the rapid maxillary expansion (RME) and also correct all transverse problems in growing patients [24]. These findings also coincide with what Cotton [25] concluded after his work with monkeys. Hicks [26] reported substantial skeletal changes with slow expansion, especially in younger children. Additionally, slow expansion is related to a more physiological reorganization of the maxilla in the three planes of the space, providing more stability and less relapse possibilities than RMEs. 

It has been observed that quad helix had significantly lower direct and indirect costs, with fewer failures requiring retreatment when compared to other expansion plates, thus concluding that quad helix is the preferred method for correcting posterior crossbite in the mixed dentition [27].

## 4. Conclusion

It should be emphasized that it is very important to correct crossbites at an early age, reducing the need for long term orthodontic therapy in the future. The case report described clearly demonstrates the versatility of using hexa helix (modified quad helix) appliance in correcting anterior and posterior bilateral crossbites. The advantages of this appliance are significant and include simple design, easy construction, minimal cost, and better results. For early treatment to be successful, the treatment timing and treatment method should exhibit proven positive results. This appliance design could help general practitioners and paediatric dentists in managing similar malocclusions.
